# The global, regional, and national burden of Invasive Non-typhoidal Salmonella (iNTS): An analysis from the Global Burden of Disease Study 1990–2021

**DOI:** 10.1371/journal.pntd.0012960

**Published:** 2025-04-01

**Authors:** Yunjuan He, Qingqing Jia, Kang Cai, Shanshan Xu, Huajun Li, Qiuling Xie, Yushu Qiu, Liya Zhang, Xianting Jiao

**Affiliations:** 1 Department of Pediatric infectious, Xinhua Hospital Affiliated to Shanghai Jiao Tong University School of Medicine, Shanghai, China; 2 Shanghai Institute of Infectious Disease and Biosecurity, Fudan University, Shanghai, China; Mohammed Bin Rashid University of Medicine and Health Sciences, UNITED ARAB EMIRATES

## Abstract

**Objectives:**

Invasive Non-typhoidal Salmonella (iNTS) can cause serious, life-threatening, and invasive infections, posing great challenges to public health. We aimed to systematically review the burden of iNTS disease based on Global Burden of Diseases (GBD) 2021.

**Methods:**

We extracted data for the incidence, death, and disability-adjusted life-years (DALYs) associated with iNTS from GBD 2021, providing an overview of its epidemiology while examining trends from 1990 to 2021. Additionally, we decomposed changes of iNTS-related burden, and quantified cross-country inequalities.

**Results:**

GBD 2021 estimated 509976(95%UI,413361 to 606167) incident cases of iNTS worldwide in 2021, with the most cases and highest age-standardized rate (ASR) in Western Sub-Saharan Africa. The low SDI region had the most cases of iNTS in 2021. The incidence and DALYs rates were highest in the Low SDI region. Among all age groups, the incidence, death, and DALYs rate of iNTS were primarily concentrated among the following age groups: <1 year, 1-4 years, 5–9 years, 10–14 years, and 15–19 years. The highest rates were observed in the <1-year group. The results of joinpoint regression analysis revealed that the global burden of iNTS increased overall from 1990 to 2005, followed by notable decrease from 2005 to 2021 at varying rates. Decomposition analysis found that population growth (103.93%) and epidemiological change (48.34%) were responsible for motivating the changes in iNTS global burden. Cross-country inequality analysis revealed that the SDI-related inequalities were moderated from 1990 to 2021.

**Conclusions:**

The global burden of iNTS is still high, and the distribution patterns vary across different countries and territories. The global burden of iNTS was primarily noteworthy among children and adolescents, with the highest burden among infants. The changes in the iNTS burden were primarily driven by population growth and epidemic transition. Despite varying iNTS burdens across different SDI regions, SDI-related inequalities across countries became moderated gradually over time. This study reported the global disease burden and temporal trends of iNTS disease, and underscores the need for age- and region-specific strategies to mitigate the corresponding global burden.

## Author summary

Invasive Non-typhoidal Salmonella (iNTS) can cause serious, life-threatening, and invasive infections, posing great challenges to public health. We have provided a comprehensive and latest evaluation of the global burden of iNTS infections based on Global Burden of Diseases (GBD) 2021. Among all age groups, the global burden of iNTS infections was most notable in childhood. The global burden of iNTS infections was more significant in the low- and low-middle SDI quintiles. Changes in the iNTS burden were primarily driven by population growth and epidemiological changes. SDI-related inequalities across countries were relieved over time. The findings underscore the substantial challenges in managing and controlling iNTS-associated diseases, notably the increasing number of cases and the global disparities in its distribution. These insights can guide the development of public health policies and effective allocation of medical resources, and highlights the imperative for the development and implementation of age- and regionally-targeted interventions to ameliorate the global iNTS-associated burden.

## Introduction

Invasive non-typhoidal Salmonella (iNTS), belong to the foodborne diseases, causes significant morbidity and mortality, with a large economic burden impacting society [1,2]. NTS infections commonly result in self-limited diarrheal enterocolitis with low case fatality. Risk factors include malnutrition, extreme age (<5 years and ≥70 years), and immunocompromised individuals, which increase susceptibility to iNTS infection [3]. When NTS organisms invade normally sterile sites, patients infected with iNTS may be subject to sepsis, meningitis, pneumonia, arthritis, and osteomyelitis [3,4]. Immunocompromised individuals, including those infected with HIV and malaria, infants and children living in areas where malnutrition is common, are particularly at risk of iNTS infection [5,6]. With a higher fatality rate than noninvasive infection, iNTS infection is a major cause of morbidity and mortality [6,7].

The latest GBD 2021 study provides data on the incidence, mortality, prevalence, and DALYs for hundreds of countries and territories worldwide, thus providing a great opportunity to compare and assess the burden and risk factors for iNTS. GBD included iNTS on its cause list in 2017[1]. With updated data and a broadly scoped systematic review, we present estimates of iNTS infection burden from GBD 2021, and execute a comprehensive assessment of the burdens of the non-typhoidal Salmonella invasive disease burden from global, regional, and national aspects. This interpretation of the GBD 2021 estimates will facilitate the development of effective preventive and control measures that can mitigate the burden of iNTS.

## 2. Methods

### Ethics statement

Informed consent was reviewed and approved by the Institutional Review Board of the University of Washington. All the information about ethical standards is available through the official website (http://www.healthdata.org/gbd).

### 2.1. Overview and data collection

The global burden of iNTS was obtained from the GBD 2021, which provides an up-to-date and comprehensive analysis about hundreds of diseases and their dozens of risk factors among 204 countries and territories. According to epidemiological similarities and geographical proximity, all countries and territories were classified into 21 regions. For purpose of this study, we utilized the GBD 2021 classification, which divides the world into 21 geographic regions based on epidemiological similarities and geographic proximity Meanwhile, all countries and territories were grouped into five categories based on the sociodemographic index (SDI; low SDI, low-middle SDI, middle SDI, high-middle SDI, and high SDI).

In this study, we collected data regarding the number of cases and incidence of iNTS, iNTS-associated mortality, and number of iNTS-associated DALYs, coupled with their corresponding rates at global, regional, and national levels. All rates were provided per 100,000 population. All GBD data are publicly available and can be downloaded in the GBD Network(http://ghdx.healthdata.org/gbd-results-tool). Herein, “Invasive Non-typhoidal Salmonella” was chosen for the cause; and “death,” “incidence,” and “disability-adjusted life years (DALYs)” for the measures from the database.

### 2.2. Descriptive analyses

Considering the uncertainty of primary data sources, data error, data manipulation and modelling uncertainty, the uncertainty for all estimates were calculated in GBD data [8]. The uncertainty was quantified and captured statistically in the 95% uncertainty intervals (UIs) for each location and for each estimate. The UIs were calculated from 1000 draw-levels from the posterior distribution of models, and 95% UIs were defined as the 2.5th and 97.5th values of the distribution. Four types of burdens, including incidence, prevalence, death, disability-adjusted life-years (DALYs), and their corresponding age-standardized rates (ASRs), were provided with 95% UIs to eliminate the effects caused by differences in population structures. The estimated annual percentage change (EAPC) and their 95% confidence intervals (CIs) were also calculated.

### 2.3. decomposition analysis

population size, age structure, and epidemiologic changes were used in a decomposition analysis to establish the relative contribution of changes in iNTS-associated disability-adjusted life-years (DALYs).

### 2.4. Joinpoint regression analysis

We used joinpoint regression analysis to detect the local trend of iNTS burden. Joinpoint regression analysis could divide the overall trend into multiple subsegments according to the inﬂection points and further evaluate the magnitude of each epidemiological trend by calculating the annual percentage change (APC) and average APC (AAPC)[9]. The conditions for using this model are: each data point is independent, and the trend is linear.

### 2.5. Cross-country inequality analysis

Health inequality monitoring can lay the groundwork for informed health planning and enhance policies, programs, and practices aimed at mitigating variations in the distribution of health outcomes. In the present study, slope index of inequality, was applied to measure the distributive inequality of iNTS burden among different SDI regions.

All analyses were performed using R software (R Core Team, version 4.4.1, Vienna, Austria) and Joinpoint software (version 5.2.0) from the Surveillance Research Program of the US National Cancer Institute. The p-value was estimated at a significance level of 0.05.

## 3. Results

### 3.1. Global trends

Globally, the case number and age-standardized rate (ASR) of incidence, death, and DALYs demonstrated dramatically increasing trends from 1990 to 2021. In 2021, there were 509,976 cases (95% UI, 413,361 to 606,167) incident cases of iNTS worldwide. The percentage of male individuals was 55% [282293, (95% UI, 228562 to 336117), higher than those of female individuals, 45% [227682, (95% UI, 184798 to 270181)]. Cases of iNTS increased by 45% (95% CI, 1.18 to -0.29) from 1990 to 2021. The corresponding incidence rate increased accordingly from 5.99(95% UI, 4.94 to 7.03) in 1990 to 7.21 per 100,000 population (95% UI 5.83 to 8.64) in 2021. (Tables 1 and S1)

**Table 1:** Global incidence and DALYs of Invasive Non-typhoidal Salmonella (iNTS) in 2021, and their EAPC of rate from 1990 to 2021.

	Incidence			DALYs		
	Number(95%UI)	Rate^a^(95%UI)	EAPC^b^(95%UI)	Number(95%UI)	Rate^a^(95%UI)	EAPC^b^(95%UI)
Global	509976(413361,606167)	7.21(5.83,8.64)	0.45(1.18,-0.29)	4740235(2762282,7597208)	69.14(39.7,111.21)	0.52(1.24,-0.2)
Sex						
Female	227682(184798,270181)	6.58(5.33,7.88)	-0.12(0.16,-0.4)	2060461(1190369,3315908)	61.71(35.36,99.94)	-1.22(-1.1,-1.34)
Male	282293(228562,336117)	7.8(6.29,9.35)	-0.47(-0.38,-0.55)	2679774(1540522,4273512)	76.15(43.75,122.16)	-1.14(-1.02,-1.26)
SDI						
High SDI	7950 (6055,10043)	0.89 (0.67,1.13)	-0.01(0.34,-0.36)	6754 (4506,10116)	0.63 (0.39,0.99)	-4.1(-3.57,-4.63)
High-middle SDI	7484 (5703,9411)	0.67 (0.51,0.85)	0.24(0.32,0.17)	17862 (9857,30947)	1.61 (0.85,2.83)	-2.13(-1.99,-2.28)
Middle SDI	57475 (46591,68867)	2.72 (2.21,3.3)	-0.03(0.44,-0.5)	237887 (129643,388178)	11.81 (6.41,19.46)	-1.07(-0.7,-1.44)
Low-middle SDI	146357 (119543,173961)	7.47 (6.13,8.84)	-0.13(0.43,-0.68)	971385 (557477,1503196)	49.6 (28.7,76.6)	-0.68(-0.18,-1.18)
Low SDI	290609 (231961,354803)	20.91 (17.08,24.83)	-1.16(-0.31,-2)	3505730 (1975759,5685368)	234.06 (135.29,376.15)	-0.84(-0.11,-1.57)
Regions						
Andean Latin America	440 (289,600)	0.67 (0.45,0.92)	-0.06(-0.01,-0.11)	1411 (726,2502)	2.19 (1.13,3.89)	-1.22(-1.1,-1.34)
Australasia	120 (74,175)	0.42 (0.25,0.62)	0.19(0.41,-0.04)	61 (47,77)	0.15 (0.12,0.2)	-2.19(0.29,-4.6)
Caribbean	217 (141,305)	0.47 (0.3,0.66)	-0.08(0.01,-0.18)	491 (220,868)	1.13 (0.51,2.02)	0.27(0.35,0.18)
Central Asia	413 (260,594)	0.43 (0.28,0.62)	0(0.14,-0.15)	384 (185,708)	0.4 (0.2,0.73)	-1.36(-1.19,-1.53)
Central Europe	415 (268,605)	0.41 (0.26,0.61)	-0.12(0.16,-0.4)	362 (247,529)	0.29 (0.19,0.43)	-6.19(-4.95,-7.41)
Central Latin America	1729 (1247,2266)	0.7 (0.51,0.9)	-1.31(-0.66,-1.96)	868 (495,1485)	0.37 (0.21,0.63)	-9.63(-7.66,-11.56)
Central Sub-Saharan Africa	44361 (35515,52842)	28.34 (23.29,33.21)	-3.54(-2.26,-4.79)	320720 (176157,511712)	198.36 (107.65,312.48)	-3.84(-2.61,-5.05)
East Asia	7088 (4693,9905)	0.52 (0.34,0.74)	-0.47(-0.38,-0.55)	24161 (12537,41199)	1.79 (0.89,3.3)	-2.86(-2.77,-2.95)
Eastern Europe	812 (522,1205)	0.46 (0.28,0.67)	-0.55(-0.22,-0.89)	878 (525,1518)	0.5 (0.3,0.82)	-2.01(-1.73,-2.28)
Eastern Sub-Saharan Africa	38990 (31939,46385)	8.28 (6.96,9.64)	-4.55(-3.95,-5.15)	311183 (178615,480394)	55.97 (32.72,86.65)	-5.07(-4.65,-5.49)
High-income Asia Pacific	764 (476,1100)	0.54 (0.3,0.8)	0.41(0.84,-0.01)	312 (150,573)	0.17 (0.07,0.35)	-2.44(-2.09,-2.78)
High-income North America	2957 (2088,3939)	0.94 (0.65,1.27)	0.03(0.43,-0.36)	1085 (961,1246)	0.25 (0.22,0.29)	-3.38(-2.2,-4.55)
North Africa and Middle East	11524 (8828,14465)	1.82 (1.41,2.28)	0.15(0.22,0.08)	64874 (34812,108743)	10.33 (5.6,17.13)	-1.14(-1.02,-1.26)
Oceania	297 (226,381)	1.91 (1.45,2.42)	0.23(0.35,0.1)	2413 (1283,4001)	15.25 (8.17,25.08)	0.05(0.22,-0.12)
South Asia	63675 (49351,78332)	3.42 (2.66,4.17)	-0.48(-0.43,-0.53)	433890 (239589,705794)	23.65 (13.17,38.2)	-1.41(-1.32,-1.51)
Southeast Asia	17080 (13103,21009)	2.6 (2.01,3.2)	-0.94(-0.87,-1.02)	81146 (44230,132819)	12.66 (6.97,20.75)	-1.98(-1.92,-2.04)
Southern Latin America	362 (240,497)	0.57 (0.37,0.78)	0.31(0.51,0.11)	11 (8,13)	0.02 (0.02,0.02)	-6.67(-5.62,-7.71)
Southern Sub-Saharan Africa	9117 (6924,12781)	11.24 (8.49,15.8)	-1.64(-1.39,-1.9)	37271 (18750,62032)	45.94 (23.24,76.53)	-2.39(-1.75,-3.03)
Tropical Latin America	1160 (798,1609)	0.55 (0.38,0.76)	-0.68(-0.39,-0.96)	2400 (1291,4087)	1.19 (0.63,2.04)	-3.07(-2.71,-3.42)
Western Europe	2496 (1742,3398)	0.76 (0.54,1.03)	1.43(1.78,1.07)	791 (678,911)	0.11 (0.09,0.12)	-8.3(-7.56,-9.04)
Western Sub-Saharan Africa	305959 (242565,375593)	47.54 (38.53,56.66)	-0.35(0.63,-1.32)	3455523 (1891891,5693979)	486.81 (266.57,789.85)	-0.35(0.49,-1.17)

^a^. The unit of the rate is per 100,000 population

^b^. EAPC is used to represent the trend of the rate. If EAPC > 0 and lower UI > 0, upward trend; if EAPC < 0 and upper UI < 0, downward trend.Otherwise, relatively stable over 30 years. Abbreviations: UI, uncertainty interval; EAPC, estimated annual percentage change; DALYs, disability–adjusted life years; SDI, Sociodemographic Index

The global number of iNTS-associated DALYs in 2021 was 4,740,235 (95% UI, 2,762282 to 7,597,208), increased by 29% from 1990 to 2021. The EAPC was 0.52(95% CI 1.24 to -0.20). The rate of DALYs associated with iNTS elevated from 1990 to 2021, 61.7(95% UI, 35.54 to 98.76) in 1990 vs 69.14(95% UI, 39.7 to 111.21) in 2021. Similarly, the number and rate of iNTS-associated death presented upward trend from 1990 to 2021.

**Fig 1:**
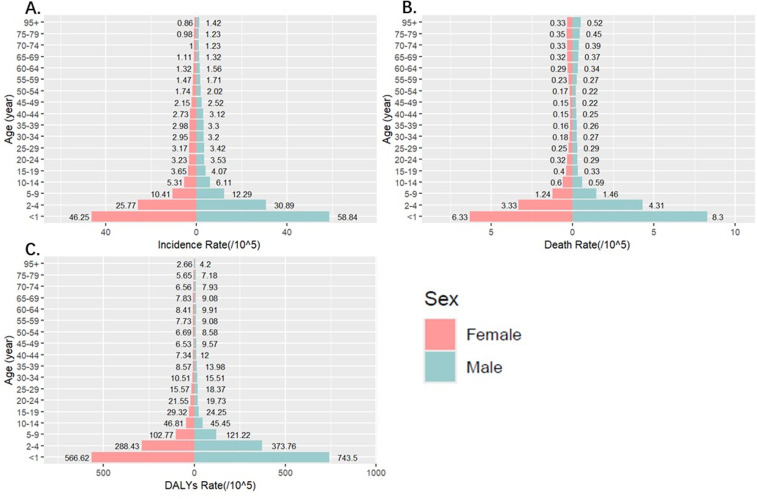
iNTS Incidence, Deaths, and Disability-Adjusted Life-Years (DALYs) at different age groups in 2021. A. age-related distributions of age-standardized incidence rate. B. age-related distributions of age-standardized death rate. C. age-related distributions of age-standardized DALYs rate.

Among all age groups, the incidence rate of iNTS was primarily concentrated among children and adolescents (Fig 1). Furtherly, we analyzed the annual trend among five age groups (<1 year, 1-4 years, 5–9 years, 10–14 years 15–19 years), from three metric of iNTS, included incidence, death and DALYs rate. It was demonstrated that children under 1 year had the highest childhood iNTS incidence rate, so as the death and DALYs. Within the age groups of less than 1 year and 1 to 4 years old, the morbidity rates initially increased and then exhibited a subsequent decline from 1990 to 2021(S1 Fig). In the context of sex, the rate of three metric associated with iNTS was more considerable among male than female.

### 3.2. SDI regional trends

**Fig 2:**
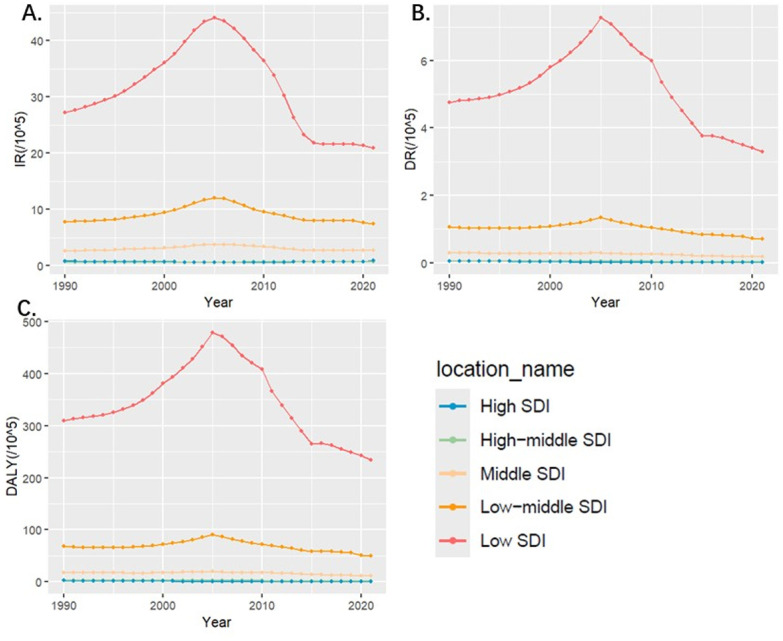
Epidemiologic Trends of Incidence, Death, and Disability-Adjusted Life-Years (DALYs) Rates in five Sociodemographic Index (SDI) Regions of iNTS from 1990 to 2021. A. Trends in incidence rate. B. Trends in death rate. C. Trends in DALYs rate.

The analysis by Socio-demographic Index (SDI) revealed significant differences in the burden of iNTS across different levels of socioeconomic development. The low SDI region had the most cases of iNTS in 2021 (290609; 95% UI, 231961 to 354803). The incidence and DALY rates were highest in the Low SDI region, with an incidence rate of 20.91 per 100,000 population (95% UI: 17.08-24.83) and a DALY rate of 234.06 per 100,000 population (95% UI: 135.29-376.15). The incident rate in the low SDI region decreased from 27.17 (95% UI,22.61 to 31.57) in 1990 to 20.91 (95% UI,17.08 to 24.83) in 2021. The EAPC was -1.16(95% CI, -0.31 to -2). In 2021, the low SDI region had the highest number of iNTS-associated DALYs (3505730; 95% UI,1975759 to 5685368) with a dramatic increase of 54% from 1990 to 2019. However, the rate of DALYs decreased from 309.46 (95% UI,173.74 to 503.05) in 1990 to 234.06 (95% UI, 135.29 to 376.15) in 2021. The EAPC was -0.84(95% CI, -0.11 to -1.57). From the scope of SDI quintiles, burden of iNTS in the low SDI region annually was much higher than other regions in terms of incidence, death and DALYs. From 1990 to 2021, the incidence rate in the low and low-middle SDI regions initially increased and then exhibited a subsequent decline, so as death and DALYs. (Fig 2)

### 3.3. Geographic regional trends

**Fig 3:**
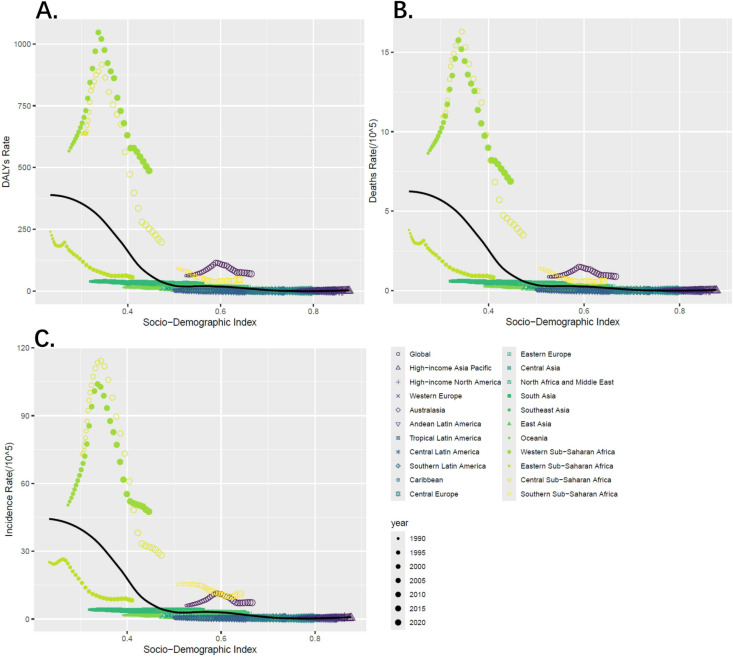
The Associations Between SDI and iNTS across 21 GBD Regions. A. Association between age-standardized DALYs rate of iNTS and SDI. B. Association between age-standardized death rate of iNTS and SDI. C. Association between age-standardized incidence rate of iNTS and SDI.

The regional analysis highlighted the significant disparities in the burden of iNTS across different parts of the world. Among 21 geographic regions, Western Sub-Saharan Africa (SDI: 0.44) had the most cases of iNTS in 2021 (305959; 95% UI,242565 to 375593), whereas Australasia (SDI: 0.84) had the fewest (120; 95% UI, 74 to 175). Western Sub-Saharan Africa also had the highest incidence rate of iNTS (47.54; 95% UI, 38.53 to 56.66), whereas central Europe (SDI: 0.79) had the fewest (0.41; 95% UI,0.26 to 0.61). From 1990 to 2021, Eastern Sub-Saharan Africa (SDI: 0.41) had the largest decrease in the incidence rate of iNTS (EAPC: -4.55; 95% CI, -3.95 to -5.15). Annually, in western and central Sub−Saharan Africa, the incidence rates were the highest level than other regions. Sub-Saharan Africa including four regions had higher rates of incidence than the global mean, whereas 17 regions had rates that were lower than the global mean. With increasing SDI, the incidence rate gradually decreased. Meanwhile, this study demonstrated that the association between DALYs rate and sociodemographic index presented similar trends, as well as death rate. (Fig 3)

### 3.4. National trends

**Fig 4:**
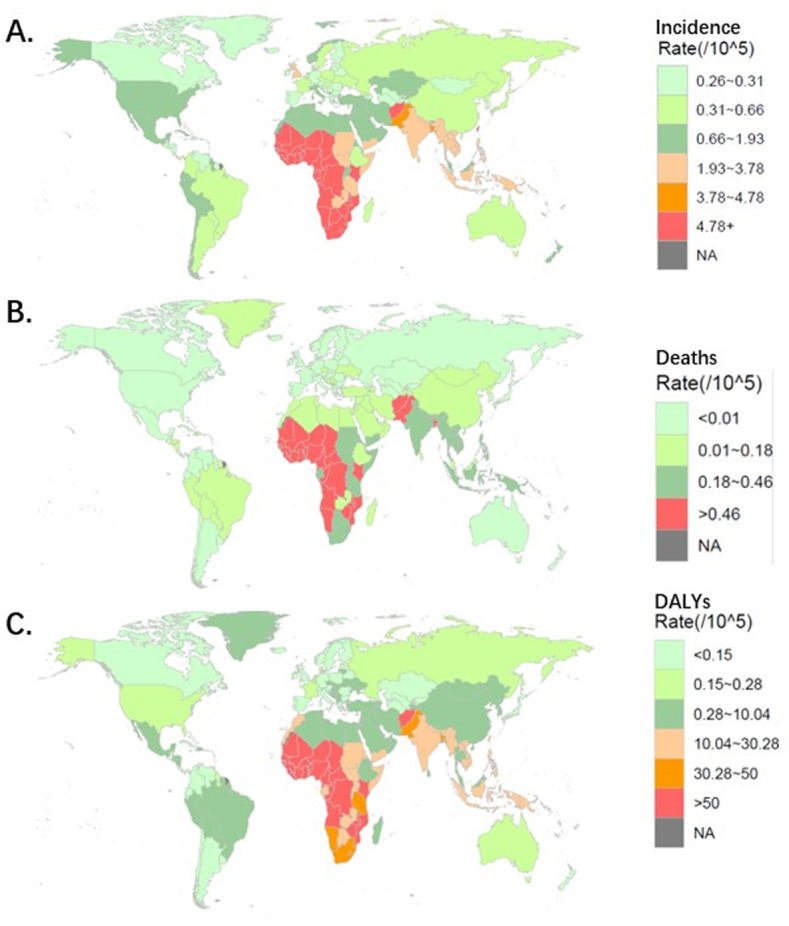
The age-standardized rate of iNTS incidence, death, DALYs in 2021 across 204 countries. A. The incidence rate of iNTS; B. The death rate of iNTS; C. DALYs rate of iNTS. Note: The basemap shapefile was from R package ‘maps’ version 3.4.2. https://cran.rstudio.com/web/packages/maps/index.html.

Nationally, the incidence, death and DALYs of iNTS varied remarkably across the world. Global burden of iNTS disease is observed to be significant in sub-Saharan Africa. In 2021, among 204 countries, Nigeria had the most numbers of iNTS-associated incidence, death and DALYs. The incidence number in Nigeria is 164230(95%UI, 201316 to 129102). The death number in Nigeria is 21614(95%UI, 35936 to 11380). The DALYs number in Nigeria is 1754289.61(95%UI, 2943184.09 to 908933.17). Mali had the highest incidence and death rate of iNTS, 107.44(95%UI, 131.61 to 85.1), 16.6(95%UI, 26.27 to 9.16), respectively. Meanwhile, Mali had the highest number of iNTS-associated DALYs, 1754289.61(95%UI, 2943184.09 to 908933.17). The global incidence rate of iNTS in 2021 was 7.21 per 100,000 population (95%UI, 5.83 to 8.64). The incidences rates were above the global mean in 32 countries and below the global mean in 172 countries. The global death rate of iNTS in 2021 was 0.88 per 100,000 population (5%UI, 0.52 to 1.41). The deaths rates were above the global mean in 27 countries and below the global mean in 177 countries. (Fig 4)

### 3.5. Burden of global iNTS using joinpoint regression analysis

**Fig 5:**
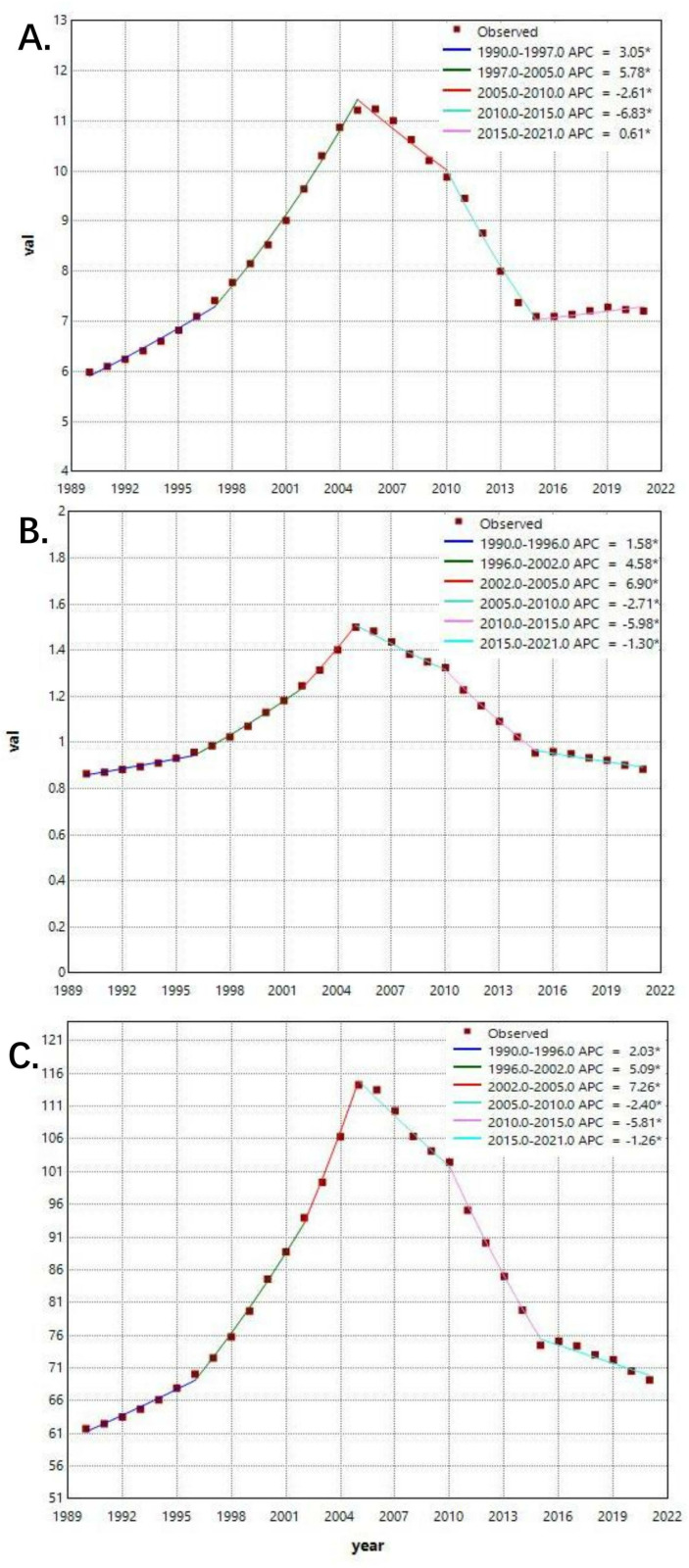
The joinpoint regression analysis on age-standardized rate of iNTS incidence, death, DALYs. A.age-standardized rate of incidence; B. age-standardized rate of death; C. age-standardized rate of DALYs. APC:annual percentage change.

The results of joinpoint regression analysis on the burden of iNTS were demonstrated in Fig 5. Between 1990 and 2021, trends for incidence rate of iNTS can be segmented five periods. The APC increased from 3.05% (1990-1997) to 5.78% (1997-2005), followed by a decline to -2.61% (2005-2010) and a more substantial decrease to -6.83% (2010-2015). The final segment from 2015 to 2021 showed a modest increase of 0.61%. Therefore, the trend for the ASR of incidence experienced four joinpoints of outstanding increases in 1990–1997, 1997–2005, followed by significant decreases in 2005-2012, 2010–2015, and slight increase in 2015-2021. Meanwhile, the trend for the ASR of death demonstrated five joinpoints of rapid increases in 1990–1996, 1996-2002 and 2002–2005, and gradual declines in 2005–2010, 2010–2015, and slight decrease in 2015–2021. The trend for DALYs rate displays a similar pattern. The trend of DALYs rate underwent five joinpoints. From 1990–2005, it kept increasing at varying rates. Followingly, a decreasing trend in 2005–2021 was exhibited.

### 3.6. Decomposition analysis on the incidence of iNTS

**Fig 6.**
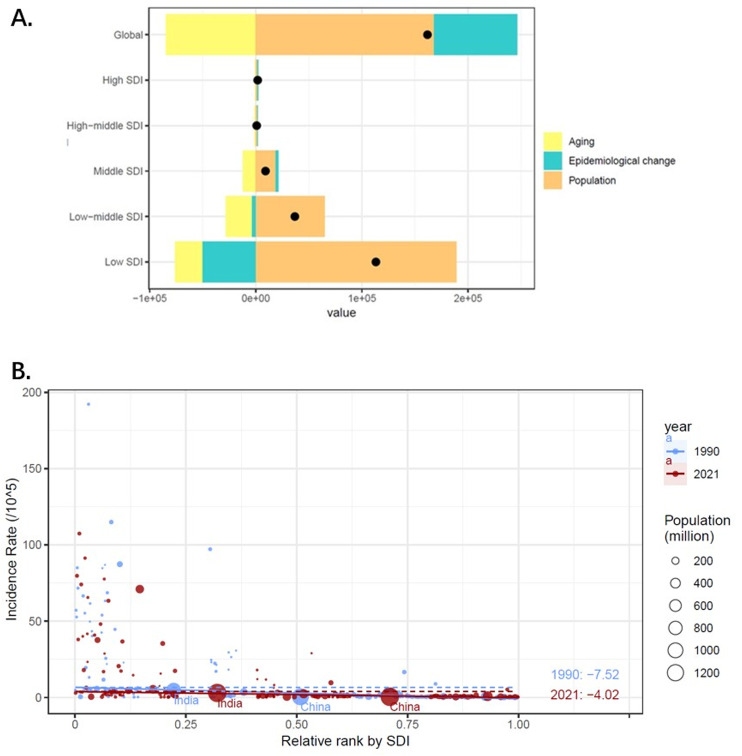
A Decomposition analysis of iNTS change in incidence by SDI, 1990 to 2021. Fig 6B. SDI-related health inequality regression for the incidence rate of iNTS worldwide, 1990 and 2021.

In the last 32 years, various evolutions occurred in age-standardized incidence rate (ASIR) of iNTS at global and different SDI regions. The most tremendous variations were detected in the low and low-middle SDI quintile. Aging, population growth and epidemiological change accounted for -52.27%, 103.93%, and 48.34% of the global increase in ASIR, respectively. The effects of demography and epidemiology on ASIR differed across subgroups. Seemingly, population growth caused the most pronounced effects on ASIR in the low and low-middle quintiles. Interestingly, the negative effects of epidemiological change on ASIR were observed in the low SDI quintile with the most pronounced presentation. (Fig 6A)

### 3.7. Cross-country inequality analysis

Slight relative SDI-associated inequalities in iNTS burden were detected, with notable increments in these inequalities occurring over time. It was demonstrated by the slope index of inequality that there was an excess of -7.52 (95% CI: -8.3 to–6.74) incidence cases per 100,000 between countries with the highest and lowest SDI in 1990, those of which declined to -4.02 (95% CI: -4.63 to –3.42) in 2021. This significant decline demonstrated a reduction in the inequality of incidence rate of iNTS among different SDI countries during this period. (Fig 6B)

## 4. Discussion

Utilizing the most recent GBD 2021 data, this study provided the latest and comprehensive insights into the incidence, death, and DALYs of iNTS infection at global, regional, and national levels from 1990 to 2021. We employed advanced statistical methods such as EAPC, decomposition analysis, and cross-country inequality analysis to evaluate temporal trends and the relative contributions of population aging, growth, and epidemiological changes. There were variations in the incidence, mortality, and DALYs of iNTS across countries. Among all age groups, the global burden of iNTS was primarily noteworthy among children and adolescents, with the highest burden among individuals aged less than 1 year. Joinpoint regression analysis revealed that the global burden of iNTS increased from 1990 to 2005, followed by a notable decrease from 2005 to 2021. Th.is trend was demonstrated to be more remarkable in low SDI and low-middle SDI regions. Decomposition analysis found that population growth and epidemiological change were major factors driving the changes in iNTS burden, especially in the low SDI quintile. Cross-country inequality analysis revealed that SDI-related inequalities lightened over time. Compared with the global burden in 1990, the ASR of incidence, prevalence, and DALYs in 2021 slightly increased. Whereas, the case number of these metrics was still eminently increasing, suggesting that a huge challenge in the control and management of iNTS remained.

By estimating the iNTS disease burden in 2010 and 2017, previous studies revealed that iNTS disease occurred with the highest incidence in sub-Saharan Africa[1,10]. As previously reported, our study emphasized that the highest number of iNTS disease cases occurred in Africa. Among 21 geographic regions, Western Sub-Saharan Africa (SDI: 0.44) had the most cases of iNTS in 2021 (305959; 95% UI,242565 to 375593). This condition may be associated with the epidemiology of iNTS in sub-Saharan Africa where some strains of S. typhimurium and S. enteritidis with high frequency of multi-drug resistant (MDR) phenotypes are circulating[11]. Conversely, Australasia (SDI: 0.84) had the fewest cases of iNTS in 2021 (120; 95% UI, 74 to 175). This disparity between Western Sub-Saharan Africa and Australasia may be attributed to endemic settings of immunocompromised diseases circulating in Western Sub-Saharan Africa, such as AIDS, malnutrition and Malaria. This hypothesis requires further research to be confirmed. Among studies on iNTS incidence by age, children and adolescents carried the highest number and rate of iNTS disease. Furthermore, children aged <5 years regularly had higher incidence rates than older children and adults, especially among infants with the highest incidence rate. Therefore, infants and younger children are key targets for iNTS vaccines[12]. Ideally, vaccines to prevent non-typhoidal Salmonella invasive disease will need to protect individuals from younger individuals and patients with host risk factors for diseases, including HIV, malaria, and malnutrition[13].

Additionally, we found significant negative correlations between SDI and iNTS disease incidence, iNTS-associated mortality rate, and iNTS-associated DALYs rate. The lightening burden in iNTS-associated disease occurred in the high and high-middle SDI regions; this condition was presumably associated with the availability of better nutritional status and medical services in the high SDI region, which allows timely diagnosis and better treatment of iNTS disease[14].

Decomposition of the iNTS incidence rate highlighted that the burden increase was primarily driven by demographic expansion epidemiological change across all development spectra. Generally, population growth tended to impose more prominent effects in lower SDI regions, suggesting a different pattern of demography in driving changes of iNTS burden in accordance with SDI[9]. Interestingly, the epidemiological change in incidence had a negative impact, but was far from offsetting the impact of population growth in the low SDI quintile. The assessment of inequalities in the burden of iNTS disease across countries based on the SDI gradient could delineate the dispersion of burdens and determine the nations requiring enhancements in iNTS preventive measures and control strategies. It’s widely assumed that in countries with high SDI, the population tends to have more extensive access to efficient healthcare systems, consequently yielding a lower disease burden. Consequently, nations globally must enhance their healthcare infrastructure to prepare for optimizing this demographic transition, placing particular emphasis on population expansion and the distribution of medical resources.

This research also encountered significant limitations. Primarily, it was heavily dependent on the GBD database, the precision of which is limited by the accessibility of national registry data. The GBD data, sourced from diverse regions and countries, exhibit variability in quality, comparability, accuracy, and the extent of missing information. Meanwhile, a limited number of countries in sub-Saharan Africa contributed the majority of the data, while several nations reported no or minimal cases due to the absence of clinical microbiological infrastructure, potentially contributing to biases[15]. Firstly, the paucity of data is primarily attributable to the challenges associated with accurate disease diagnosis, which necessitates well-equipped microbiology laboratories and trained technicians—resources that are often difficult to sustain in settings with limited resources[2]. Furthermore, clinic-based surveillance systems are likely to miss individuals with iNTS disease who do not seek medical care. While iNTS disease is generally considered a severe illness prompting healthcare utilization, milder or moderate forms may exist and go unreported. Since we relied on passive surveillance data, the actual incidence of iNTS infections is likely much higher, with the reported cases likely representing the most severe instances. Immunocompromised individuals, such as those with HIV or malaria, as well as infants and young adults residing in areas with high rates of malnutrition, are particularly susceptible to iNTS disease [16]. Recent studies have suggested a positive correlation between the incidence of iNTS disease and risk factors such as the incidence rate of Plasmodium falciparum, HIV prevalence, and the prevalence of underweight individuals [14]. In this study, we did not account for potential changes in the epidemiology of HIV, which may have influenced the global burden of iNTS disease.

In summary, as a major public health issue, the incidence, death and DALYs of iNTS varied considerably across countries, experienced outstanding increase firstly from 1990 to 2005, and then prominent decrease from 2005 to 2021. Although EAPCs in iNTS-associated incidence rate and DALYs rate have demonstrated minimal variation worldwide, the number of iNTS-associated incidence continued to rise obviously. The global burden of iNTS was primarily noteworthy among children and adolescents, with the highest burden among infants. Considering that children and adolescents constitute the major groups subject to iNTS infections, interventions such as vaccination and better nutrition status will need to be executed among younger individuals urgently. The changes in iNTS burden were primarily driven by population growth and epidemic transition. Despite varying iNTS burdens across different SDI regions, the SDI-related inequalities across countries became moderated gradually over time. The findings underscore the substantial challenges in managing and controlling iNTS-associated diseases, notably the increasing number of cases and the global disparities in its distribution. These insights can guide the development of public health policies and effective allocation of medical resources. To enhance personalized healthcare systems and address the distinct medical needs of each country, global health policymakers should contemplate targeted interventions and adaptable strategies.

## Supporting information

S1 Table Global Incidence, Deaths and DALYs of Invasive Non-typhoidal Salmonella (iNTS) in 1990 and 2021 (DOCX)

S1 Fig Trends in iNTS Incidence, Deaths, and Disability-Adjusted Life-Years (DALYs) Among Children and Adolescents From 1990 to 2021 (TIFF)
