# Supplementary material for: The global, regional, and national burden of Invasive Non-typhoidal Salmonella (iNTS): An analysis from the Global Burden of Disease Study 1990–2021
Source: PLoS Negl Trop Dis. 2025 Apr 1;19(4):e0012960. doi: 10.1371/journal.pntd.0012960 (PMC11977977; doi:10.1371/journal.pntd.0012960)
Supplement: S1 Table — (DOCX) [file pntd.0012960.s001.docx]

**The global, regional, and national burden of Invasive Non-typhoidal Salmonella (iNTS):**

**An analysis from the Global Burden of Disease Study 1990-2021.**

**Supplementary appendix**

**S1 Table. Global Incidence, Deaths and DALYs of Invasive Non-typhoidal Salmonella (iNTS) in 1990 and 2021.**

|  | **1990number(95%UI)** | | |  | **2021number(95%UI)** | | |  | **1990Rate*(95%UI)** | | |  | **2021Rate*(95%UI)** | | |
| --- | --- | --- | --- | --- | --- | --- | --- | --- | --- | --- | --- | --- | --- | --- | --- |
|  | **Incidence** | **Deaths** | **DALYs** |  | **Incidence** | **Deaths** | **DALYs** |  | **Incidence** | **Deaths** | **DALYs** |  | **Incidence** | **Deaths** | **DALYs** |
| Global | 348244(284325,411890) | 48941(28140,77609) | 3665717(2104403,5883901) | | 509976(413361,606167) | 62018(36079,97786) | 4740235(2762282,7597208) | | 5.99(4.94,7.03) | 0.86(0.5,1.36) | 61.7(35.54,98.76) | | 7.21(5.83,8.64) | 0.88(0.52,1.41) | 69.14(39.7,111.21) |
| **Sex** |  |  |  |  |  |  |  |  |  |  |  |  |  |  |  |
| Female | 159018(129682,189183) | 22066(12873,35135) | 1653384(953965,2691529) | | 227682(184798,270181) | 27164(15723,43238) | 2060461(1190369,3315908) | | 5.58(4.59,6.6) | 0.79(0.46,1.26) | 56.92(32.99,92.17) | | 6.58(5.33,7.88) | 0.79(0.46,1.26) | 61.71(35.36,99.94) |
| Male | 189226(154082,223038) | 26875(15266,42237) | 2012333(1128989,3225388) | | 282293(228562,336117) | 34854(20187,54864) | 2679774(1540522,4273512) | | 6.38(5.28,7.47) | 0.94(0.54,1.45) | 66.32(37.32,106.06) | | 7.8(6.29,9.35) | 0.97(0.56,1.54) | 76.15(43.75,122.16) |
| **Regions** |  |  |  |  |  |  |  |  |  |  |  |  |  |  |  |
| High SDI | 6177 (4852,7676) | 508 (421,622) | 19205 (14391,26038) | | 7950 (6055,10043) | 216 (160,303) | 6754 (4506,10116) | | 0.76 (0.6,0.94) | 0.05 (0.04,0.07) | 2.39 (1.76,3.27) | | 0.89 (0.67,1.13) | 0.01 (0.01,0.02) | 0.63 (0.39,0.99) |
| High-middle SDI | 6650 (5157,8399) | 579 (345,916) | 34406 (19330,56883) | | 7484 (5703,9411) | 402 (227,673) | 17862 (9857,30947) | | 0.64 (0.5,0.8) | 0.06 (0.03,0.09) | 3.37 (1.93,5.48) | | 0.67 (0.51,0.85) | 0.03 (0.02,0.05) | 1.61 (0.85,2.83) |
| Middle SDI | 48328 (38987,60268) | 4828 (2860,7225) | 330923 (196686,507400) | | 57475 (46591,68867) | 3652 (2024,5785) | 237887 (129643,388178) | | 2.59 (2.13,3.19) | 0.29 (0.18,0.43) | 17.68 (10.58,26.63) | | 2.72 (2.21,3.3) | 0.17 (0.09,0.27) | 11.81 (6.41,19.46) |
| Low-middle SDI | 109560 (89658,130461) | 13589 (7822,20692) | 1003834 (584552,1571525) | | 146357 (119543,173961) | 13408 (7733,20745) | 971385 (557477,1503196) | | 7.78 (6.48,9.1) | 1.05 (0.6,1.6) | 68.04 (39.26,104.03) | | 7.47 (6.13,8.84) | 0.71 (0.41,1.09) | 49.6 (28.7,76.6) |
| Low SDI | 177428 (142892,212904) | 29426 (16498,47655) | 2276461 (1275558,3751446) | | 290609 (231961,354803) | 44331 (25755,71309) | 3505730 (1975759,5685368) | | 27.17 (22.61,31.57) | 4.77 (2.67,7.42) | 309.46 (173.74,503.05) | | 20.91 (17.08,24.83) | 3.3 (1.91,5.19) | 234.06 (135.29,376.15) |
| Andean Latin America | 279 (183,383) | 21 (11,34) | 1395 (751,2306) | | 440 (289,600) | 25 (13,44) | 1411 (726,2502) | | 0.66 (0.44,0.9) | 0.06 (0.03,0.09) | 3.2 (1.75,5.16) |  | 0.67 (0.45,0.92) | 0.04 (0.02,0.07) | 2.19 (1.13,3.89) |
| Australasia | 72 (43,105) | 3 (2,3) | 84 (72,98) |  | 120 (74,175) | 3 (2,4) | 61 (47,77) |  | 0.37 (0.23,0.54) | 0.01 (0.01,0.02) | 0.44 (0.38,0.52) | | 0.42 (0.25,0.62) | 0.01 (0,0.01) | 0.15 (0.12,0.2) |
| Caribbean | 166 (112,235) | 6 (3,11) | 406 (190,755) |  | 217 (141,305) | 8 (4,15) | 491 (220,868) |  | 0.46 (0.32,0.65) | 0.02 (0.01,0.03) | 1.07 (0.51,1.97) | | 0.47 (0.3,0.66) | 0.02 (0.01,0.03) | 1.13 (0.51,2.02) |
| Central Asia | 304 (190,441) | 7 (4,11) | 478 (271,772) |  | 413 (260,594) | 6 (3,12) | 384 (185,708) |  | 0.42 (0.27,0.61) | 0.01 (0.01,0.01) | 0.58 (0.33,0.92) | | 0.43 (0.28,0.62) | 0.01 (0,0.01) | 0.4 (0.2,0.73) |
| Central Europe | 486 (315,686) | 60 (51,69) | 2616 (2266,3025) | | 415 (268,605) | 14 (10,19) | 362 (247,529) |  | 0.41 (0.27,0.58) | 0.05 (0.05,0.06) | 2.57 (2.24,2.94) | | 0.41 (0.26,0.61) | 0.01 (0.01,0.01) | 0.29 (0.19,0.43) |
| Central Latin America | 1784 (1363,2242) | 291 (266,319) | 16167 (14613,17982) | | 1729 (1247,2266) | 17 (10,27) | 868 (495,1485) |  | 1.06 (0.82,1.33) | 0.25 (0.23,0.27) | 9.75 (8.89,10.73) | | 0.7 (0.51,0.9) | 0.01 (0,0.01) | 0.37 (0.21,0.63) |
| Central Sub-Saharan Africa | 49068 (39002,58683) | 6465 (3643,10613) | 475507 (263698,782514) | | 44361 (35515,52842) | 4578 (2498,7234) | 320720 (176157,511712) | | 72.67 (60.75,84.62) | 10.94 (6.04,17.53) | 633.55 (355.04,1022.84) | | 28.34 (23.29,33.21) | 3.5 (1.92,5.48) | 198.36 (107.65,312.48) |
| East Asia | 7223 (4747,10387) | 909 (483,1540) | 53752 (27456,93022) | | 7088 (4693,9905) | 590 (304,1047) | 24161 (12537,41199) | | 0.59 (0.4,0.83) | 0.08 (0.04,0.14) | 4.48 (2.36,7.63) | | 0.52 (0.34,0.74) | 0.04 (0.02,0.06) | 1.79 (0.89,3.3) |
| Eastern Europe | 1263 (802,1825) | 37 (24,56) | 1876 (1242,2831) | | 812 (522,1205) | 23 (14,40) | 878 (525,1518) |  | 0.6 (0.38,0.88) | 0.02 (0.01,0.02) | 0.95 (0.65,1.41) | | 0.46 (0.28,0.67) | 0.01 (0.01,0.02) | 0.5 (0.3,0.82) |
| Eastern Sub-Saharan Africa | 59292 (48396,70924) | 8796 (4878,14362) | 684464 (376864,1128372) | | 38990 (31939,46385) | 4031 (2352,6238) | 311183 (178615,480394) | | 25.27 (21.42,29.48) | 3.82 (2.15,6) | 239.95 (133.55,389.02) | | 8.28 (6.96,9.64) | 0.83 (0.48,1.29) | 55.97 (32.72,86.65) |
| High-income Asia Pacific | 531 (287,824) | 18 (12,26) | 721 (420,1242) |  | 764 (476,1100) | 10 (5,18) | 312 (150,573) |  | 0.33 (0.18,0.51) | 0.01 (0.01,0.02) | 0.46 (0.27,0.79) | | 0.54 (0.3,0.8) | 0 (0,0.01) | 0.17 (0.07,0.35) |
| High-income North America | 1714 (1271,2214) | 80 (73,88) | 2505 (2333,2715) | | 2957 (2088,3939) | 44 (38,52) | 1085 (961,1246) | | 0.64 (0.47,0.81) | 0.02 (0.02,0.03) | 0.88 (0.82,0.95) | | 0.94 (0.65,1.27) | 0.01 (0.01,0.01) | 0.25 (0.22,0.29) |
| North Africa and Middle East | 7363 (5634,9329) | 951 (524,1488) | 65873 (36236,105538) | | 11524 (8828,14465) | 1002 (550,1647) | 64874 (34812,108743) | | 1.86 (1.42,2.34) | 0.28 (0.15,0.42) | 16.15 (8.86,25.35) | | 1.82 (1.41,2.28) | 0.17 (0.09,0.28) | 10.33 (5.6,17.13) |
| Oceania | 129 (99,164) | 16 (9,27) | 1095 (592,1873) | | 297 (226,381) | 35 (19,57) | 2413 (1283,4001) | | 1.69 (1.3,2.1) | 0.26 (0.14,0.43) | 14.35 (7.71,24.09) | | 1.91 (1.45,2.42) | 0.26 (0.14,0.42) | 15.25 (8.17,25.08) |
| South Asia | 53685 (41905,67096) | 7141 (3806,11537) | 511933 (270751,835272) | | 63675 (49351,78332) | 6598 (3683,10608) | 433890 (239589,705794) | | 4.14 (3.26,5.07) | 0.6 (0.32,0.95) | 38.51 (20.54,62.36) | | 3.42 (2.66,4.17) | 0.37 (0.21,0.59) | 23.65 (13.17,38.2) |
| Southeast Asia | 18539 (14589,22473) | 1873 (1046,2934) | 123963 (69078,196681) | | 17080 (13103,21009) | 1451 (806,2356) | 81146 (44230,132819) | | 3.58 (2.82,4.3) | 0.43 (0.24,0.66) | 23.87 (13.32,37.2) | | 2.6 (2.01,3.2) | 0.23 (0.12,0.37) | 12.66 (6.97,20.75) |
| Southern Latin America | 245 (168,341) | 1 (1,1) | 51 (44,59) |  | 362 (240,497) | 0 (0,0) | 11 (8,13) |  | 0.49 (0.34,0.68) | 0 (0,0) | 0.1 (0.09,0.12) |  | 0.57 (0.37,0.78) | 0 (0,0) | 0.02 (0.02,0.02) |
| Southern Sub-Saharan Africa | 9084 (6487,15710) | 790 (401,1505) | 58047 (28829,118565) | | 9117 (6924,12781) | 482 (249,805) | 37271 (18750,62032) | | 15.18 (11.32,24.07) | 1.42 (0.77,2.51) | 90.66 (45.96,174.23) | | 11.24 (8.49,15.8) | 0.6 (0.31,1.01) | 45.94 (23.24,76.53) |
| Tropical Latin America | 1073 (745,1402) | 91 (65,130) | 6011 (4133,8743) | | 1160 (798,1609) | 46 (26,76) | 2400 (1291,4087) | | 0.66 (0.48,0.86) | 0.07 (0.05,0.09) | 3.79 (2.69,5.34) | | 0.55 (0.38,0.76) | 0.02 (0.01,0.03) | 1.19 (0.63,2.04) |
| Western Europe | 1675 (1147,2293) | 221 (199,245) | 4839 (4475,5264) | | 2496 (1742,3398) | 49 (40,57) | 791 (678,911) |  | 0.46 (0.31,0.63) | 0.04 (0.04,0.05) | 1.12 (1.04,1.21) | | 0.76 (0.54,1.03) | 0 (0,0.01) | 0.11 (0.09,0.12) |
| Western Sub-Saharan Africa | 134271 (106888,163958) | 21165 (12014,34432) | 1653933 (932929,2708928) | | 305959 (242565,375593) | 43006 (23552,69967) | 3455523 (1891891,5693979) | | 50.48 (41.21,59.46) | 8.64 (4.89,13.47) | 566.79 (320.48,918.78) | | 47.54 (38.53,56.66) | 6.88 (3.91,10.95) | 486.81 (266.57,789.85) |

*The unit of the rate is per 100,000 population；

Abbreviations: DALYs: Disability-Adjusted Life Years；UI: uncertainty interval.
