# Supplementary figures and images for: The global, regional, and national burden of Invasive Non-typhoidal Salmonella (iNTS): An analysis from the Global Burden of Disease Study 1990–2021
Source: PLoS Negl Trop Dis. 2025 Apr 1;19(4):e0012960. doi: 10.1371/journal.pntd.0012960 (PMC11977977; doi:10.1371/journal.pntd.0012960)

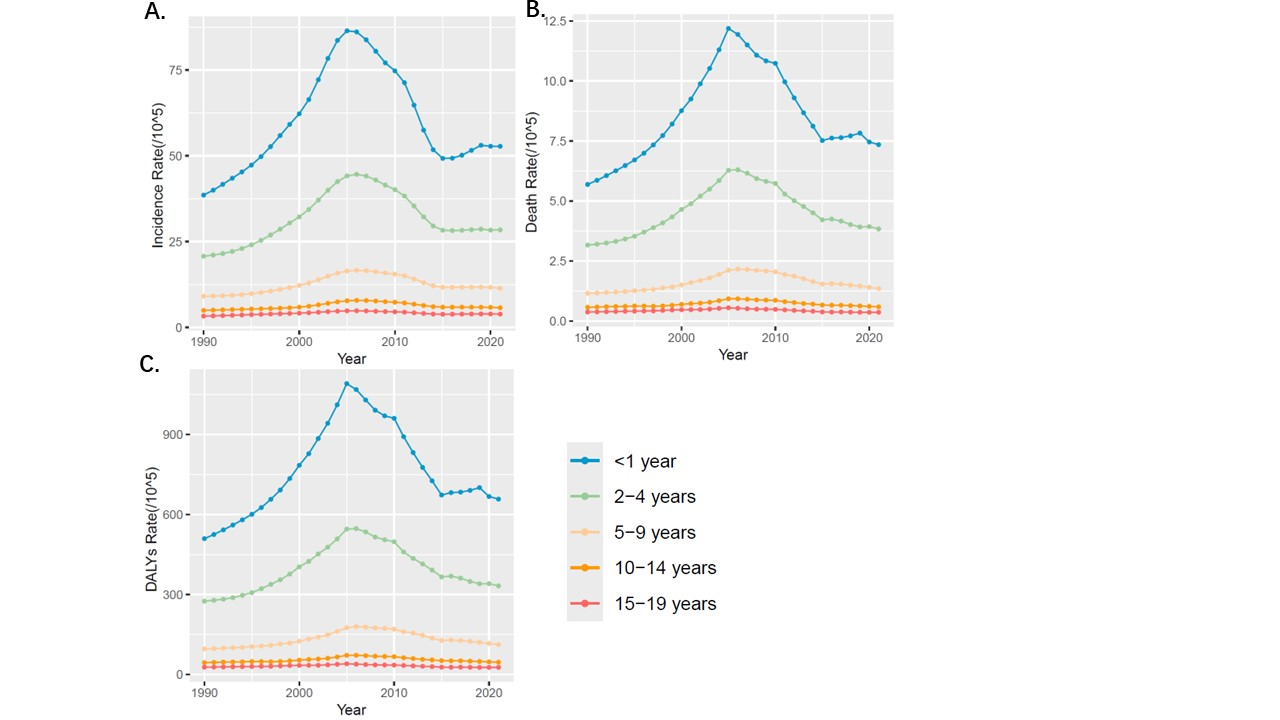

Supplement: S1 Fig — (TIFF) [file pntd.0012960.s002.tiff]
